# Assessment of depression and suicidal behaviour among medical students in Portugal

**DOI:** 10.5116/ijme.57f8.c468

**Published:** 2016-10-29

**Authors:** Ricardo Coentre, Carlo Faravelli, Maria Luísa Figueira

**Affiliations:** 1 Faculty of Medicine, University of Lisbon, Lisbon, Portugal; 2 Department of Health Sciences, Psychology and Psychiatry Unit, University of Florence, Florence, Italy

**Keywords:** Medical students, depression, suicidal behaviour, Portugal

## Abstract

**Objectives:**

To examine depression and suicidal behaviour and associated factors in a sample of medical students in Portugal.

**Methods:**

We conducted a cross-sectional study design of 456 native Portuguese medical students from the 4^th^ and 5^th^ year at the University of Lisbon. Participants answered a self-report survey including questions on demographic and clinical variables. Statistical analyses were conducted using the chi-square test, with a Monte Carlo simulation when appropriate.

**Results:**

Depression among medical students was 6.1% (n=28) and suicidal behaviour 3.9% (n=18). Higher depression scores were noted in female medical students (χ^2^=4.870,df=2,p=0.027), students who lived alone (χ^2^=8.491,df=3,p=0.037), those with poor physical health (χ^2^=48.269,df=2,p<0.001), with poor economic status (χ^2^=8.579,df=2,p=0.014), students with a psychiatric diagnosis (χ^2^=44.846,df=1,p=0.009), students with a family history of psychiatric disorders (χ^2^=5.284,df=1,p=0.022) and students with high levels of anxiety (χ^2^=104.8, df=3, p<0.001).  Depression scores were also higher in students with suicidal ideation (χ^2^=85.0,df=1,p<0.001), suicidal plan (χ^2^=47.9,df=1,p<0.001) and suicidal attempt (χ^2^=19.2,df=1,p<0.001). Suicidal behaviour was higher in medical students who lived alone (χ^2^=16.936,df=3,p=0.001), who had poor physical health (χ^2^=18,929,df=2,p=0.001), poor economic status (χ^2^=9.181,df=2,p=0.01), who are/were in psychopharmacology treatment (χ^2^=30.108,df =1,p<0.001), and who had high alcohol use (χ^2^=7.547,df=2,p=0.023), severe depression (χ^2^=88.875,df=3,p<0.001) and high anxiety levels (χ^2^=50.343,df=3,p<0.001). The results also revealed that there were no differences between students in the 4^th^ and 5^th^ years of medical school regarding rate of depression and suicidal behaviour.

**Conclusions:**

Since depression and suicidal behaviour are mental health problems affecting a significant proportion of medical students, medical schools should implement programs that promote mental health wellness, physical health and economic status between other factors.

## Introduction

In addition to medical doctors, medical students have higher rates of depression and suicidal behaviour compared to age-matched general population.^1^^-^^6^ The rate of depression in medical students ranged from 2.9% to 38.2%.^7^^,^^8^ Studies suggest that depression is higher in female medical students,^6^^,^^9^^-^^14^ younger students^15^^-^^17^ and students in their early years in medical school.^12^^,^^17^^-^^19^ Mixed results have been obtained regarding the relationship between depression and ethnicity.^1^^,^^4^^,^^11^^,^^20^

Medical students have academic, psychological and existential stressors. Additionally, school selection may favour individuals with perfectionism, altruist traits and self-critical or performance-based self-esteem, which predispose individuals to depression and suicidal behaviour.^11^ The consequences of untreated depression include substance use, school dropout, poor academic performance and negative repercussions on long-term patient care (more cynical, less empathetic and less willing to care for chronically ill patients).^7^^,^^21^

Major depression is the most significant antecedent risk factor for suicide, but other factors, including other mood disorders, substance abuse, hopelessness, adverse life events, personal history of physical or sexual abuse, and family history of suicide, often play a role.^22^ Suicide is a major public health challenge. Rates of suicide among physicians are high.^23^^,^^24^ In addition to knowledge and easy access to means of suicide, these high rates of suicide in physicians also reflect the effects of untreated psychological symptoms in medical students.^25^^,^^26^ Suicidal ideation in medical students ranged from 4.4% to 23.1%, and suicidal attempts from 0.0% to 6.4%.^7^ Suicide is an extreme consequence of mental health problems, so it is critical for medical schools to identify students at the greatest risk for suicide in the hope of intervening before a tragic outcome.^27^ Some studies have been published about depression, but there have been few studies about suicidal behaviour in medical students.

Studies have found that inadequate treatment is significant amongst depressive medical students.^1^^,^^28^ Barriers to seeking mental health care among medical students include concerns about time, confidentiality, stigma, and the potential negative effects on their careers.^28^^,^^29^ Moreover, medical students who report moderate to severe depression are much more likely to endorse the opinion that a stigma is associated with depression compared with their non-depressed colleagues.^9^ This is the first research study to investigate depression and suicidal behaviour in a sample of Portuguese medical students.

The aims of the present study were: 1) to determine the rates of depression and suicidal behaviour in a sample of 4^th^ - and 5^th^ -year of native Portuguese medical students; 2) to compare rate of depression and suicidal behaviour to general population and with medical students from other countries; 3) to investigate the relationships between personal, epidemiological and clinical factors and depression and suicidal behaviour in medical students. We hypothesised that the rate of depression and suicidal behaviour in medical students would be similar to or higher than that of the general Portuguese population. Our hypothesis is that female students, students who lived alone, students with poor economic status, students with poor physical health, students with a personal or family psychiatric history, and students with high levels of anxiety and substance and alcohol use have higher prevalence of depression. We hypothesised that male students, students who lived alone, students with poor economic status, students with poor physical health and students with high levels of depression, alcohol use and anxiety have higher rate of suicidal behaviour. We also hypothesised that medical students in their 4^th^ year of medical school have a higher prevalence of depression and suicidal behaviour than 5th year medical students as a result of the stress related to beginning of their clinical years.  

## Methods

### Participants

A cross-sectional survey was conducted in 4^th^ and 5^th^ year native Portuguese medical students of Faculty of Medicine, University of Lisbon, in Portugal, between October 2012 and July 2013. Survey responses were anonymous. Non-Portuguese medical students were excluded from the survey.

### Procedures

The study was approved by the Ethics Committee of Faculty of Medicine, University of Lisbon. Questionnaires and consent forms were given to each student. The questionnaires were distributed during psychiatric lessons, which have an approximate duration of 120 minutes. We surveyed 10 classes during the 10-month duration of the research, three from the 4^th^ year and seven from the 5^th^ year of medical school. We used the last 20 minutes of each lesson to administer the survey.  Psychiatric lessons are mandatory for all 4^th^ and 5^th^ year medical students. Student participation in our study was voluntary, and those who agreed to participate signed the consent form, completed the survey and submitted it separately to ensure confidentiality. Students who refused to participate submitted incomplete surveys in the same manner.

### Instruments

A paper questionnaire was used. We thought that a paper questionnaire rather than an online questionnaire warranted a higher response rate without significantly compromising confidentiality and anonymity. The questionnaire included six sections with a total of 95 questions: personal data (25 questions), substance use (one question), suicidal behaviour (three questions), alcohol use (25 questions), depression (21 questions) and anxiety (20 questions). The questionnaires were printed in self-administered forms based on the evidence that the self-report responses of potentially embarrassing behaviours (e.g., suicidal behaviour) were higher in self-administered questionnaires than in an interviewer-administered format.^30^

### Depression

The Beck Depression Inventory (BDI) was used to assess depressive symptoms.^31^ It is a 21-question, multiple-choice, self-report inventory used to evaluate the incidence and severity of depression symptoms. Scores of 0 to 13 indicate no or minimal depression, 14 to 19 indicate mild depression, 20 to 28 indicate moderate depression, and 29 to 63 indicate severe depression. The version of the BDI scale that had been translated into Portuguese language and validated for the Portuguese population was used.^32^^,^^33^

**Table 1:** Demographic and personal characteristics of the study participants (N=456)

Variable		N	%
Gender			
	Male	152	33.3
	Female	304	66.7
Age (Years), M = 23.35 (SD = 3.02)			
	20 or 21	40	8.8
	22	184	40.4
	23	139	30.5
	24 or 25	44	9.6
	26 to 30	29	6.4
	more than 30	20	4.4
Year in Medical School			
	Fourth	133	29.2
	Fifth	323	70.8
Race			
	Caucasian	453	99.3
	Black	1	0.2
	Indian	2	0.4
Civil Status			
	Single	422	92.5
	Married	32	7.0
	Divorced	1	0.2
	Widow	1	0.2
Household			
	Alone	74	16.2
	Original family	208	45.6
	Girl/boyfriend/ husband/wife	36	7.9
	Friends/ colleagues	138	30.3
Smoker			
	No	398	87.3
	Yes	58	12.7
Previous University Degree			
	No	408	89.5
	Yes	48	10.5
Physical Health			
	Poor	7	1.5
	Average	114	25.0
	Good	335	73.5
Economic Status			
	Poor	11	2.4
	Average	204	44.8
	Good	240	52.7
Belief in God			
	No	198	43.4
	Yes	258	56.6
Speciality intend to choose			
	Medical specialties	148	32.5
	Surgical specialties	109	23.9
	Psychiatry	11	2.4
	General Practitioner	36	7.9
	Paediatrics	46	10.1
	Obstetrics and Gynaecology	37	8.1
	Unknown	50	11.0
	Ophthalmology/ Otorhinolaryngology(ORL)	15	3.3
	Others	4	0.9
	Total	456	100

### Suicidal behaviour

Suicidal behaviour was assessed using a questionnaire of dichotomous questions (yes/no). Suicidal behaviour was defined as suicidal ideation (“During medical school, have you ever seriously thought about committing suicide?”), suicide plan (“During medical school, have you ever made a plan for committing suicide?”) and/or suicide attempt (“During medical school, have you ever attempted suicide?”). Students were considered to have suicidal behaviour if one of the answers was positive. This assessment was based on inventories developed by Meehan and colleagues and Lee and colleagues, which were used in other studies intended to assess suicidality in medical students and in other epidemiologic studies.^30^^,^^34^

### Personal data

This questionnaire included questions about demographic characteristics, trichotomous questions for physical health:  “What is your general level of physical health?” and economic status: “What is your general level of economic status?” This questionnaire also included an open question about future specialty intent: “What specialty do you think you will choose?” This section included questions about personal and familial psychiatric history, namely, past/current personal psychiatric diagnosis, past/current psychopharmacological treatment, history of personal psychiatric admission, and family psychiatric diagnosis. There was also a question asking if there was a suicide attempt in a close personal relationship, followed by a question identifying the relationship. Family history of psychiatric disorder and suicidal behaviour are well known risk factors to psychiatric disorders and suicidal behaviour.^35^^,^^36^

### Anxiety symptoms

For the evaluation of anxiety symptoms, the Zung Self-Rating Anxiety Scale (SAS) was used.^37^ The SAS is a self-rated scale consisting of 20 items. It is a four-point scale (rated 1-4) in which severity is assessed based on the combination of intensity, frequency and duration of symptoms. Some of the items are reversely scored. An index for the scale is derived by dividing the sum of the values (raw scores) obtained on the 20 items by the maximum possible score of 80, converting to a decimal and multiplying by 100. The scores range from 20 to 80, where 20-44 is the normal range, 45–59 indicates mild to moderate anxiety levels, 60–74 indicates marked to severe anxiety, and 75–80 indicates extreme anxiety. The version that had been translated into Portuguese and validated for the Portuguese population was used.^38^

### Alcohol use

Alcohol use was evaluated using The Michigan Alcoholism Screening Test (MAST), which has been internationally validated for the assessment of alcohol abuse.^39^ It is a 25-item self-report questionnaire, which includes questions concerning various problems associated with alcohol use, including medical, interpersonal and legal consequences. The total MAST score can range from 0 to 53, with item weights that range from 0 to 5. A score of three points or less was considered non-alcoholic, a score of four points was suggestive of alcoholism, and a score of five points or more indicated alcoholism. A validated and translated version of the scale to the Portuguese language and population was used.^40^

### Substance use

Substance use was assessed using the multi-choice question “During the last 12 months have you used the following substance(s)?” The answer included five options: cannabis/marijuana, cocaine, heroin, stimulants or amphetamines and sedatives or hypnotics not prescribed by a doctor.

**Table 2:** Depression rate by demographic and clinical characteristics

Variable		Depression BDI≥14		p-value
		N	%	
Gender				
	Male	4	2.6	0.027^*^
	Female	24	7.9	
Age Year				
	20 or 21	2	5.0	0.190
	22	15	8.2	
	23	3	2.2	
	24 or 25	5	11.4	
	26 to 30	2	6.9	
	more than 30	1	5.0	
Year in Medical School				
	Fourth	7	5.3	0.617
	Fifth	21	6.5	
Civil Status				
	Single	27	6.4	0.751
	Married	1	3.1	
	Divorced	0	0.0	
	Widow	0	0.0	
Household				
	Alone	9	12.2	0.037^*^
	Original family	9	4.3	
	Girl/boyfriend/husband/wife	0	0.0	
	Friends/ colleagues	10	7.2	
Smoker				
	No	24	6.0	0.797
	Yes	4	6.9	
Physical Health				
	Poor	4	57.1	< 0.001^†^
	Average	15	13.2	
	Good	9	2.7	
Economic Status				
	Poor	2	18.2	0.014^*^
	Average	18	8.8	
	Good	8	3.3	
Belief in God				
	No	12	6.1	0.950
	Yes	16	6.2	
Specialty Intend To Choose				
	Medical	7	4.7	0.416
	Surgical	4	3.7	
	Psychiatry	1	9.1	
	GP	3	8.3	
	Paediatrics	5	10.9	
	Obstetrics	1	2.7	
	Unknown	6	12.0	
	Ophthalmology/ORL	1	6.7	
Psychiatric Disorder				
	No	13	3.3	< 0.001^†^
	Yes	15	25.9	
Psychiatry Diagnosis				
	Depressive Disorder	7	19.4	0.009^†^
	Anxiety Disorder	2	15.4	
	Other	4	57.1	
	Bipolar Disorder	2	100	
Current/Past Psychopharmacology				
	No	15	3.7	< 0.001^†^
	Yes	13	26.5	

*Statistically significant at p<0.05. †Statistically significant at p<0.01; BDI: Beck Depression Inventory.

### Data analysis

Data were analysed using IBM SPSS (Statistical Package for the Social Sciences) Statistics software version 21. Prevalence and summary statistics were calculated on all variables of interest. BDI, SAS and MAST scale scores were collapsed into respective categories mentioned. Continuous variables are shown as the mean and standard deviation (SD). Using the chi-square test (5% level of significance), we compared the prevalence of depression and suicidal behaviour by several socio-demographic and clinical variables. When more than 20% of cells showed an expected frequency of less than 5, as the maximum value can only be 20%, the chi-square test with the Monte Carlo simulation was used. This was based on generating random samples, which overcome the problem of classes with few or no observations. In these cases, the p-value analysed was obtained by the Monte Carlo simulation.

## Results

### Demographics

A total of 456 of the 459 surveyed medical students participated in this study (response rate: 99.3%). The total number of medical students enrolled in the 4^th^ and 5^th^ years of medical school was 767, approximately 50 students in each class. Table 1 provides complete demographic details and personal characteristics of the responding students.

A total of 58 (12.7%) students had a psychiatric diagnosis, 36 (7.9%) had a depressive disorder, 13 (2.9%) had anxiety disorder, two (0.4%) had bipolar disorder, and seven (1.5%) had another psychiatric diagnosis (eating disorders, OCD, etc.). Seven (1.5%) participants had a previous psychiatric admission. Forty-nine (11%) of the medical students were or are in psychopharmacologic treatment.

In the sample, 168 (37%) students had a psychiatric family history, and 51 (11.2%) had a suicide attempt in a close relation. Twelve (2.6%) medical students had a suicide attempt in parents, two (0.4%) in a sister/brother, four (0.9%) in a boy/girlfriend, five (1.1%) in a friend and 28 (6.1%) in another close relation.

### Depression

The overall rate of depression was 6.1%, with mild depression (14≤BDI≤19) found in 14 (3.1%) of the students and moderate depression (20≤BDI≤28) found in 11 (2.4%). Three (0.7%) of the students had BDI scores ≥29, placing them in the severe depression category. Four hundred and twenty-eight (93.9%) medical students had BDI scores in the range of 0 to 13, indicating no or minimal depression. Table 2 shows the prevalence of depression by demographic and clinical characteristics.

Depression (BDI score ≥14) was higher in female medical students (χ^2^= 4.870, df=2, p=0.027), in those students who lived alone (χ^2^=8.491, df=3, p=0.037), in those with poor physical health (χ^2^=48.269, df=2, p<0.001) and in those with poor economic status (χ^2^=8.579, df=2, p=0.014). A higher prevalence of depression was also found in students who had a psychiatric diagnosis (χ^2^=44.846, df=1, p=0.009), who have current or past psychopharmacological treatment (χ^2^=39.604, df=1, p<0.001), who have been diagnosed with bipolar disorder (χ^2^=10.823, df=3, p=0.009) and who have a psychiatric family history (χ^2^=5.284, df=1, p=0.022).

The percentage of depression was higher for the category “extreme anxiety levels”, followed by “marked to severe anxiety levels” and lower for the category “normal range”, as measured by SAS (χ^2^=104.8, df=3, p<0.001). Depression prevalence was higher in students with suicidal ideation (χ^2^=85.0, df=1, p<0.001), suicide plan (χ^2^=47.9, df=1, p<0.001) or suicide attempt (χ^2^=19.2, df=1, p<0.001).

**Table 3:** Suicidal behaviour rate by demographic characteristics

Variable		Suicidal Behaviour		p-value
		N	%	
Gender				
	Male	7	4.6	0.610
	Female	11	3.6	
Age Years				
	20 or 21	1	2.5	0.927
	22	7	3.8	
	23	7	5.0	
	24 or 25	2	4.5	
	26 to 30	1	3.4	
	more than 30	0	0.0	
Year in Medical School				
	Fourth	4	3.0	0.508
	Fifth	14	4.3	
Civil Status				
	Single	17	4.0	0.986
	Married	1	3.1	
	Divorced	0	0.0	
	Widow	0	0.0	
Household				
	Alone	9	12.2	0.001^†^
	Original Family	4	1.9	
	Girl/boyfriend/husband/wife	0	0.0	
	Friends/colleagues	5	3.6	
Smoker				
	No	18	4.1	0.217
	Yes	0	0.0	
Previous University Degree				
	No	18	4.4	0.138
	Yes	0	0.0	
Physical Health				
	Poor	2	28.6	0.001^†^
	Average	9	7.9	
	Good	7	2.1	
Economic Status				
	Poor	2	18.2	0.010^*^
	Average	11	5.4	
	Good	5	2.1	
Belief in God				
	No	9	4.5	0.566
	Yes	9	3.5	
Specialty Intend to Choose				
	Medical	5	3.4	0.446
	Surgical	2	1.8	
	Psychiatry	1	9.1	
	GP	2	5.6	
	Paediatrics	3	6.5	
	Obstetrics	2	5.4	
	Unknown	1	2.0	
	Ophthalmology/ORL	2	13.3	
	Others	0	0.0	

*Statistically significant at p<0.05. †Statistically significant at pp<0.01.

### Suicidal behaviour

The rate of suicidal behaviour during medical school was 3.9% in the sample. Seventeen (3.7%) students answered yes to the question about suicidal ideation, five (1.1%) answered yes to the suicide plan question and three (0.7%) answered yes to the suicide attempt question. Medication overdose was the method used in the three medical students who had attempted suicide. Suicidal behaviour was higher in students who had poor physical health (χ^2^=18,929, df=2, p=0.001) or a poor economic status (χ^2^=9.181, df=2, p=0.01) and in students who lived alone (χ^2^=16.936, df=3, p=0.001). Suicidal behaviour was also higher in those participants who had a psychiatric diagnosis (χ^2^=23.461, df=1, p<0.001), who have current/past psychopharmacology treatment (χ^2^=30.108, df =1, p<0.001) and who have been diagnosed with bipolar disorder (χ^2^=13.321, df=3, p=0.008). The prevalence of suicidal behaviour was higher in students with a family history of psychiatric disorder (5.4% versus 3.1%), but the differences were not statistically significant (χ^2 ^= 1.394, df=3, p=0.238). Table 3 provides the prevalence of suicidal behaviour by demographic characteristics.

Suicidal behaviour was higher for the category “alcoholism” and lower for the category “non-alcoholism” in the MAST scale (χ^2^=7.547, df=2, p=0.023). The percentage of suicidal behaviour was also higher for the category “severe depression” and lower for the category “no or minimal depression” in the BDI score (χ^2^=88.875, df=3, p<0.001). In regards to anxiety symptoms measured by SAS the percentage of suicidal behaviour was higher for the category “marked to severe anxiety levels” and lower for the category “normal range” (χ^2^=50.343, df=3, p<0.001). Because there is only one case in the extreme category, its comparison is not relevant (Table 4).

**Table 4:** Suicidal behaviour by alcohol use, depression and anxiety scales score

Category		Suicidal Behaviour		p-value
		N	%	
Alcohol Use (MAST score)				
	No apparent problem	8	2.4	0.023^*^
	Suggestive of alcoholism	4	7.4	
	Alcoholism	6	8.5	
Depression (Beck Depression Inventory Score)				
	No or minimal depression	8	1.9	< 0.001^†^
	Mild depression	4	28.6	
	Moderate depression	4	36.4	
	Severe depression	2	66.7	
Anxiety (Zung Self-Rating Anxiety Scale Score)				
	Mild to moderate anxiety levels	6	7.4	
	Marked to severe anxiety levels	4	50.0	
	Extreme anxiety levels	0	0.0	
	Normal range	8	2.2	< 0.001^†^

*Statistically significant at p<0.05. †Statistically significant at p<0.01.

### Anxiety symptoms

The SAS results revealed a mean value score of 38.12 (dispersion of 21.5%). In the sample, 366 (80.3%) of the participants had a score in the normal range (score 20-44), 81 (17.8%) had mild to moderate anxiety levels (score 45-59), eight (1.8%) had marked to severe anxiety levels (score 60-74), and one (0.2%) had an extreme anxiety level (score 75-80).

### Alcohol use

Three hundred and thirty-one (72.6%) of the respondents had a MAST score ≤3, placing them in the non-alcoholic group. Fifty-four (11.8%) students had a MAST score of 4, suggestive of alcoholism, and 71 (15.6%) had a MAST score ≥5, indicative of alcoholism.

### Substance use

In the sample, 143 (31%) students admitted to substance use in the last 12 months. Of these, 92 (64%) used cannabis, 37 (27%) used sedatives not prescribed by a doctor, three (2.1%) used cannabis and stimulants, three (2.1%) used cocaine and sedatives, three (2.1%) used cannabis and sedatives, two (1.4%) used heroin, one (0.7%) used  stimulants, and one (0.7%) used cannabis, cocaine and stimulants.

The results also revealed that there were no differences between students in the 4^th^ and 5^th^ years of medical school regarding alcoholism (p=0.944), depression (p=0.672), anxiety (p=0.897) and suicidal behaviour (p=0.508).

## Discussion

This is the first Portuguese study investigating depression and suicidal behaviour in medical students. Even in Europe, this is one of the few studies that address suicidal behaviour in medical students.

The response rate of 99.3% renders an adequate sample of the population studied. We think that completing the questionnaires during classes contributed to the high response rate obtained.

Previous studies showed that depression prevalence for medical students is similar or higher than that for the general population.^1^^-^^6^ Comparison with the Portuguese general population is difficult because of the scarcity of studies. Somewhat surprisingly, the prevalence found in our current research is in line with (not higher than) the rate of depression in the general population. The only major study evaluating the prevalence of depression in Portugal found a similar prevalence to ours (6.8%) for major depressive disorders in the general population.^51^ Unexpectedly, this study in a representative sample of the Portuguese general population found high rates of prevalence of mental health disorders, only lower than those of Northern Ireland. In this research, Portuguese rates are more in line with those of northern European countries than those of southern European countries, where Portugal is geographically located.^52^ Some methodological limitations of this study could contribute to the high rates of mental disorders observed.

Previous studies in other countries that used BDI to measure depression in medical students yielded similar results (5-35.1%) to the current research.^1^^,^^5^^,^^10^^,^^13^^-^^15^^,^^53^^-^^58^ Recent, high-quality studies have reported a lower prevalence of depression that previous studies.^59^ Further, in the current research, a conservative cut-off for depression in the BDI scale (14) was used that surely contributed to a lower rate of depression in our sample of medical students.

Depression and suicidal behaviour are determined by multiple factors, including personal, biological and genetic factors, as well as social and cultural factors.^41^^-^^44^ These latter factors contribute to variation in the prevalence of depression and suicidal behaviour across different cultural contexts.^45^^-^^47^ There is evidence that cultural factors influence the cultural variation in the prevalence of depression. Cultural differences in stress, standards of living, unemployment, stigma and reporting bias are among those factors.^48^

Published research shows that, on average, the lifetime prevalence of depression is higher in high-income than in low-to middle-income countries.^48^ For example, the highest rates were in France, the Netherlands, New Zealand and the USA. It is also well recognized that people from southern Europe, where Portugal is located, have low rates of depression compared to those in other European countries.^49^ Independent of individual level effects, more depressive symptoms were recorded in countries with greater income inequality and with less individualistic cultures. Personal circumstances and beliefs and the mentioned cultural factors may all contribute to depressive symptoms.^50^ In the only study that compared the prevalence of depression in medical students from different cultures, the highest prevalence was found in the Middle East, followed by North America, Asia and South America, and the lowest rates were observed in Europe.^8^ Taking all of these factors into account, it was expected that the prevalence of depression in our research would be in the lower limits of previous studies of depression in medical students. In line with these results, our findings demonstrate that Portuguese medical students experience depression frequently, but in the lower range of published studies in other countries. Based on findings from previous studies, we could speculate that individualistic culture, average socio-economic background and stigma associated to mental health disorders are factors that explained our observed incidence of depression in medical students.

Another significant finding of this study was a gender difference regarding the association with depression, where female students reported a significantly higher prevalence of depression than male students. This gender variation in the depressive status in medical students could be a reflection of the usual trend of higher prevalence of depression in females in the general population.^51^

Certain risk factors are found to be widely associated with depression in the general population, such as smoking, economic status and psychiatric diagnosis.^52^^,^^53^ This study partially confirms these findings in medical students, with depression being higher in students who reported poor economic status and a psychiatric diagnosis. Regarding smoking, the prevalence of depression was higher in the medical students who smoked, but with no statistically significant difference.

Anxiety symptoms are frequently observed with depression,^43^^,^^54^ so it is not surprising that depression was higher in those medical students with higher SAS scores.

How the depression rate changes over the years of medical school is not completely clear. Some longitudinal studies suggested that the depression rate is high during the first year, followed by a gradual decline during the later years of medical school.^55^^,^^56^ In the first year of medical school, factors such as incomplete adaptation to a new environment, loneliness and difficulty in establishing a close relationship with other people could explain the high rates of depression in this phase of medical school.^57^ Other studies indicate that the first year of medical training and the beginning of the clinical phase, when entering the wards (in the Portuguese system, this occurs in the fourth year), are associated with the highest rates of depression.^20^Patients´ suffering, teachers´ criticisms, neglect from patients and their families could contribute to the high levels of depression in the fourth year of medical school.^7^ Current research included 4^th^  and 5^th^ year medical students in order to compare the prevalence of suicidal behaviour and depression in the first year of the clinical phase of their medical degree (fourth year in the Portuguese medical curriculum) and another year (fifth year). We did not find any significant differences in depression prevalence between the fourth year and fifth year of medical school, so we could not conclude that depression is higher in first year of the clinical phase in Portuguese medical students.     

Suicidal behaviour (suicidal ideation, suicide plan and/or suicide attempts) is a precursor to the final outcome of suicide, which may be preventable if the signs are detected early.^35^ To prevent such occurrences, there is a need to understand the extent of the problem in various populations and cultures, along with its epidemiology.

There is a considerable cross-national variability in the prevalence of suicidal behaviours in the general population.^58^ Interestingly, the prevalence of suicidal behaviour does not mirror the geographic pattern of suicide death. Namely, there are high rates in eastern Europe, a middle to low prevalence in southern Europe (where Portugal is located) and low rates in South America.^58^ Additionally, there are no significant differences between developed and developing countries.^59^ Suicidal behaviour in the present study is lower than that in the study of Jeon et al. in South Korea (23.1%), which used similar questions to our study. However, in this last study, lifetime suicidal behaviour was investigated, not just medical school time as in the current research.^60^ The rate of suicidal ideation observed in our research was similar to the Schwenk et al. (4.4%) US study^9^ and the Irish study by Curran et al. (5.9%).^61^ Regarding suicide planning, comparison is not possible due to the lack of studies investigating it as an outcome. Suicide attempts found during medical school was higher than that observed in other studies, such as in an Austrian sample (0.3%) 62 or in the Swedish study, where no suicide attempts in the last 12 months were reported.^3^ Because suicide attempts are an important predictor of suicide,^22^^,^^35^ we could speculate that this high rate of suicide attempts in medical students corresponds to a high rate of suicide in medical students. Unfortunately, there are no published statistics regarding completed suicides in Portuguese medical students to confirm this theory.

The current study found higher rates of suicidal behaviour in medical students who lived alone, who had poor physical health and who had a poor economic status. Contrary to the general population, where it is well demonstrated that females are overrepresented in nonfatal suicidal behaviour and men in complete suicide, which is known as the “gender paradox of suicidal behaviour”,^63^^,^^64^ there was a higher rate of suicidal behaviour in male students (but with no significant differences). This is contrary to the known increased risk of suicidal ideation, as well as suicide completion, in female physicians.^23^ As with depression, there was no relation between suicidal behaviour and year of medical school. Suicidal behaviour was higher but not significantly different in medical students who had a family history of psychiatric disorder and who had a suicide attempt in a close relation (the majority referred to family members). This is concordant with the well-documented fact that a family history of suicidal behaviour is a risk factor for suicidal behaviour.^36^

As expected, suicidal behaviour was higher in students in the categories suggestive of alcoholism or alcoholism on the MAST scale, depressive categories on the BDI and marked or severe anxiety level categories in the SAS. This suggests that suicidal behaviour is present in students who have clinically relevant mental disorders. In our sample, suicidal behaviour was higher in conjunction with bipolar disorder, followed by depressive disorders and other psychiatric disorders. This is concordant with previous data in the general population indicating that affective disorders are the leading mental disorders associated with suicide.^35^

One interesting finding of the current study was that in spite of the lack of statistical significance, suicidal behaviour was higher in medical students who intend to choose psychiatry and ophthalmology/otorhinolaryngology in the future. This is concordant with the known data that among physicians, psychiatrists are considered to be at a greater risk of suicide, followed by ophthalmologists and anaesthesiologists.^65^

Comparisons between the prevalence of depression and suicidal behaviour in Portuguese medical students and other Portuguese university students are difficult due to the paucity of studies in both populations. There are no published studies about depression or suicidal behaviour in Portuguese non-medical university students. Studies from other countries have found high rates of depression in other university students, namely, business, nursing and pharmacy students.^10^^,^^11^^,^^61^^,^^66^ One possible explanation is that medical students maintain this high prevalence of depression and suicidal behaviour after completing their medical degree and throughout their professional lives, making the medical profession one of the professions with the highest rates of depression and suicidality.^23^^,^^67^

### Implications of the findings

Medical students are future doctors and need to be protected from preventable causes of morbidity and mortality, such as depression or suicidal behaviour. Medical schools should have a system to identify students who are currently depressed or suicidal. Schools should also work to identify students at risk of future mental health disorders. Some reports mention simple, brief tools to identify medical students who are at risk of mental disorders.^68^ However, despite the availability of safe and effective treatment, depressed medical students are undertreated. Additionally, those who reported suicidal ideation are not more likely to receive treatment for their mental disorders.^1^ For these reasons, simply identifying medical students at risk it is not sufficient. Students at risk or with identified mental disorders must be treated. Providing easy access to mental health care, ideally outside the faculty, maintaining anonymity and reducing the stigma related to seeking mental health treatment are important factors to be addressed.^69^ Medical students should have access to psychiatry appointments in a confidential and anonymous way. 

It is recommended that medical schools implement programs and curriculum modifications to contribute to lowering mental health problems. However, it is unrealistic to completely eliminate factors (e.g., stress) associated with mental health problems. All medical schools should implement student wellness programs to promote and provide resources for healthy living mainly in the 1st year and probably recycle them in the beginning of the clinical years of medical school. These programs could include curricular changes, stress management, psychoeducation, coping strategies, mindfulness programs, individual counselling and managing financial stressors and alcohol use.^6^^,^^69^^-^^75^ As our results illustrate, the promotion of physical health (e.g., promoting physical exercise, easier access to general health services) and economic status (e.g., expanding scholarships) should be included in these programs once both are associated with depression and suicidal behaviour.

Finally, we could speculate that by promoting mental health in medical students and treating those who need it, we are also diminishing the known high rates of suicide in medical doctors.    

### Limitations of the study

This cross-sectional study had several methodological limitations. Student concerns about the confidentiality of a study conducted by members of their faculty could have influenced students´ responses. Additionally, a cross-sectional design of the study was used rather than a longitudinal one, which is more compelling to establish causality between the associations found. Another limitation is that the study was conducted at a single institution. The use of self-reported measures rather than clinical structured interviews could also be considered a limitation. Depending on personality traits, students may exaggerate or ignore their symptoms. The occurrence of recall bias and reporting errors is also possible. However, the survey design ensured anonymity, and students completed the questionnaire voluntarily, which should have promoted honest responses. This also permits access to a larger sample of participants, which constitutes a clear advantage. Furthermore, the current study did not control the time of completing questionnaires by medical students. For example, we could speculate that near exam periods, scores on the anxiety scale would be higher. Finally, the use of the non-validated scales to some variables, namely, to physical health, economic status and substance use was also a limitation.

To overcome some of the above-mentioned limitations, future research should focus on longitudinal research and involve staff not belonging to the medical school in order to provide more robust confidentiality to medical students. Clarifying some of our non-significant findings will also be important, namely, the relationships between depression and alcoholism, gender and suicidal behaviour and speciality and suicidal behaviour.    

## Conclusions

This is the first study of depression and suicidal behaviour in a sample of Portuguese medical students. This study confirmed that depression and suicidal behaviour are mental health problems affecting a significant proportion of medical students, but at a rate similar to the general Portuguese population. Similar to the general Portuguese population, our research found rates of depression among medical students in the lower limit of published studies in other countries. Depression was higher in female medical students, students who lived alone, students with poor physical health, students with a poor economic status, students with a psychiatric diagnosis, students with a family history of psychiatric problems and students with high levels of anxiety. Depression was also higher in students with suicidal ideation, suicide plans or suicide attempt.

We found quite alarming rates regarding suicidal behaviour, namely suicide attempts. Suicidal behaviour was higher in medical students who lived alone, students who had poor physical health, students who had a poor economic status, students who are/were in psychopharmacology treatment and students who had high alcohol use, severe depression and high anxiety levels. Medical schools should implement programs that promote mental health wellness, physical health and economy economic status between other modifiable factors associated.

### Acknowledgements

We would like to thank all medical students who participated and completed the survey. 

### Conflict of Interest

The authors declare that they have no conflict of interest.
